# Pyrrolotriazinone as an Underexplored Scaffold in Drug Discovery

**DOI:** 10.3390/ph14121275

**Published:** 2021-12-06

**Authors:** Tony Ge, Jean-Christophe Cintrat

**Affiliations:** Département Médicaments et Technologies pour la Santé (DMTS), SCBM, Université Paris-Saclay, CEA, INRAE, 91191 Gif-sur-Yvette, France; mstonyge@gmail.com

**Keywords:** pyrrolotriazinone, heterocycle, inhibitor, antagonist

## Abstract

Heterocyclic amino derivatives have been extensively synthesized and validated as potent bioactive compounds, and nowadays, numerous marketed drugs share these scaffolds, from very simple structures (monoamino, monocyclic compounds) to much more complex molecules (polycyclic derivatives with two or more nitrogen atoms within the (fused) rings). In a constant quest for new chemical entities in drug discovery, a few novel heterocycles have emerged in recent years as promising building blocks for the obtainment of bioactive modulators. In this context, pyrrolotriazinones have attracted attention, and some show promising biological activities. Here, we offer an extensive review of pyrrolo[2,1-f][1,2,4]triazin-4(1H)-one and pyrrolo[1,2-d][1,2,4]triazin-4(3H)-one, describing their biological properties en route to drug discovery.

## 1. Introduction

Whereas biologics are emerging as a new concept in the therapeutic arsenal, small molecules are still needed either to identify probes to decipher biological processes or to get potential starting points for drug discovery. For many reasons (synthetic accessibility, dogma in medicinal chemistry), therapeutically useful scaffolds are limited. Looking at the vastness of chemical space, one can easily understand that only a tiny part of this universe has been explored so far. As an example, in 2010, all possible molecules of putative interest for drug discovery with a molecular weight below 500 g/moL were estimated to reach 10^60^ [1]. So, even if the chemical libraries available are growing, there are still missing or at least underrepresented chemical families. This can be partly explained by the difficulties in synthesizing scaffolds that are not compatible with the criteria required for the construction of chemical libraries (few steps, robust and high yielding chemistry). As a consequence, unprecedented scaffolds (with structural novelty) are rarely selected as starting points for drug design, even if this would be highly desirable, especially when facing new target classes such as DNA, RNA, and protein/protein interfaces. Hence, the repertoire of available scaffolds for drug discovery remains limited even if fragment-based approaches [2] can overcome this limitation. In order to narrow the number of scaffolds to explore, one can focus on nitrogen-containing compounds and, more precisely, on nitrogen-based heterocycles. This makes sense since more than 75% of drugs approved by the Food and Drug Administration currently available on the market (β-lactams, quinazolines, benzodiazepines) are nitrogen-containing heterocycle moieties. In addition, it is noteworthy that in 2014 the average number of nitrogen atoms per drug was 2.3 N/drug for all the small-molecule drugs, while it reached 3.1 N/drug in those containing a nitrogen heterocycle. In this case, looking at heterocycles containing more than one nitrogen atom seems appropriate [3,4,5]. The structural features of nitrogen-based heterocycle derivatives are highly favorable in drug discovery since they may exhibit broad bioactivities and improved water solubility compared to oxygen-based heterocycles. Whereas many nitrogen-based heterocycles have been explored in drug design, there are still some families that are underrepresented [6].

**Figure 1:**
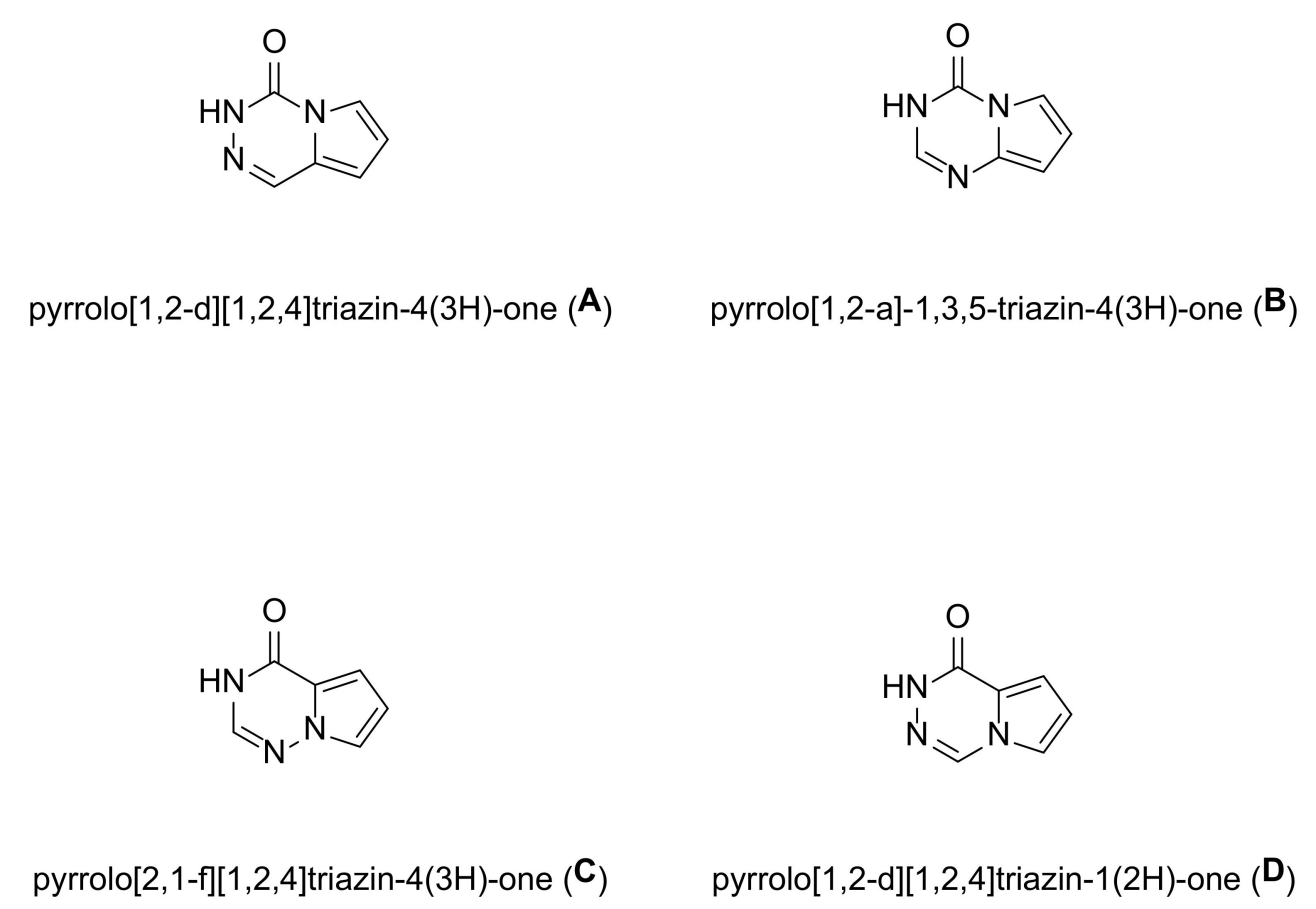
General structure of pyrrolotriazinones.

In this review, we will pay particular attention to pyrrolotriazinones. Pyrrolotriazinones, a class of azolotriazinone, are indeed a fused bicyclic compound. Pyrrolotriazinones share common properties with all nitrogen-containing heterocycles. The electron-rich nitrogen heterocycle can establish various weak interactions with a target based on hydrogen bonding, dipole–dipole interactions, hydrophobic effects, van der Waals forces, and π-stacking. Herein, we will restrict to those members in which the nitrogen atom of the pyrrole ring is also part of the triazinone moiety. Of note is that a few isomers can fall into this class of compounds (something which depends on the positions of the nitrogen atoms within the triazinone ring) such as pyrrolo[1,2-a]-1,3,5-triazin-4(3H)-one, pyrrolo[1,2-d][1,2,4]triazin-4(3H)-one, pyrrolo[2,1-f][1,2,4]triazin-4(1H)-one, or pyrrolo[1,2-d][1,2,4]triazin-1(2H)-one, Figure 1 below. Herein, we will focus on pyrrolo[2,1-f][1,2,4]triazin-4(1H)-one, even though a few other analogs, for instance pyrrolo[1,2-d][1,2,4]triazinone, have also demonstrated encouraging biological activities in various therapeutic areas [7]. Of course, other analogs may “virtually” exist, but either their synthesis is too complex, or they lead to unstable compounds, and they will therefore not be taken into account in this review. Whereas no drugs belonging to this class of compounds are marketed yet, there is no doubt that the biological activities demonstrated so far by these compounds may pave the way for future drugs.

**Scheme 1:**
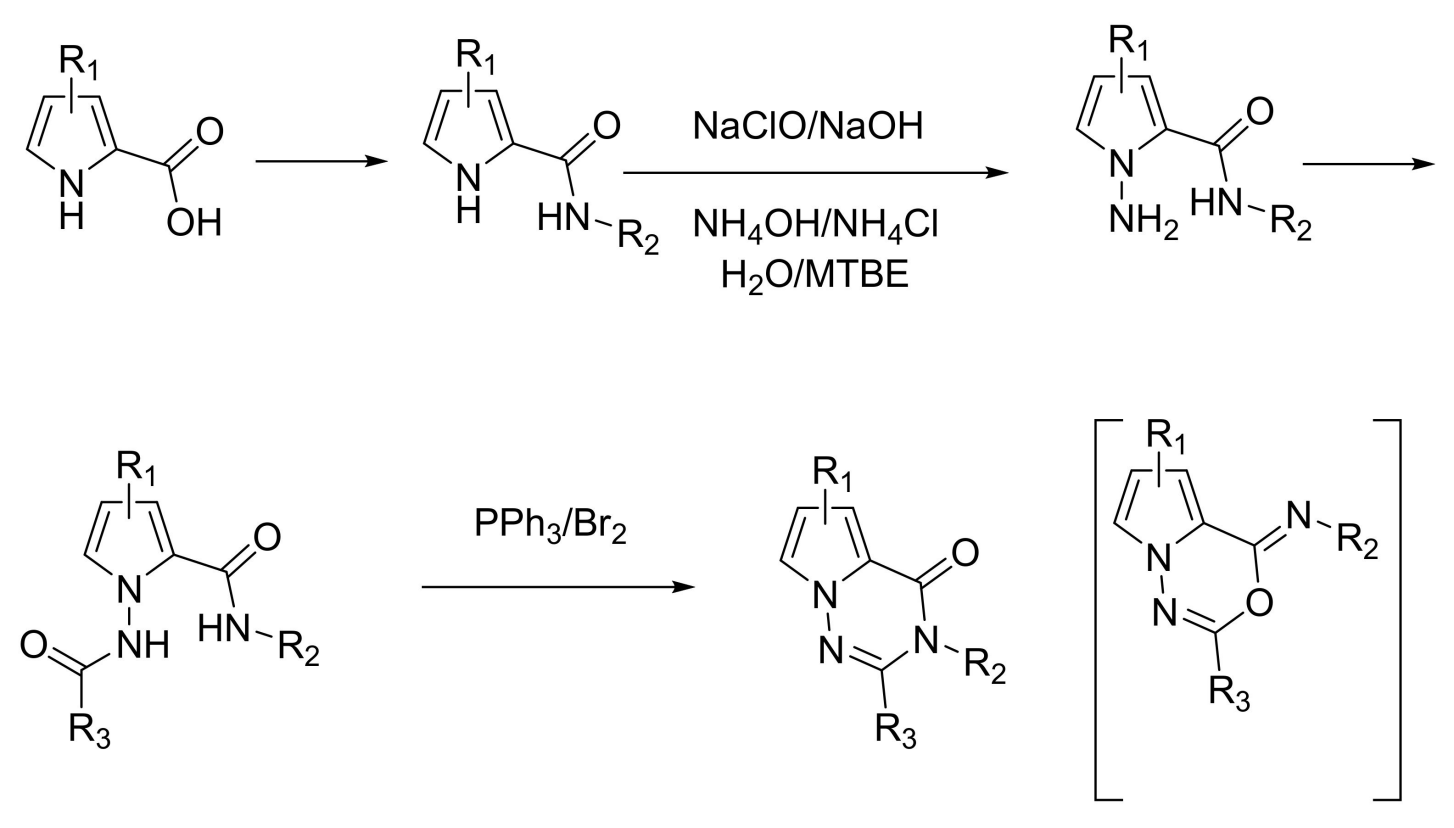
General route towards pyrrolotriazinones.

Because of the very recent interest in pyrrolotriazinones and their biological applications, the synthetic routes are scarce. Specifically in regard to the synthesis of pyrrolo[2,1-f][1,2,4]triazin-4(1H)-one (pyrrolotriazinone throughout the article), all the routes rely on the same general strategy. The starting material, substituted pyrrole-2-carboxylic acid, is first converted into an amide based on conventional peptide coupling methodologies or via amidation of the corresponding acyl chloride. Then, the pyrrole is converted into 1-amino-pyrrole (with in situ generated chloramine under phase transfer condition) that is further acylated and the corresponding product is cyclized under oxidative conditions thanks to PPh_3_/Br_2_ into the title compound according to Scheme 1 [8].

Due to the growing interest in biological applications of pyrrolotriazinones, new synthetic strategies should be explored in the future in order to offer a quicker access to more complex analogs, a highly challenging task for organic chemists.

As mentioned above, only a few articles deal with either the synthesis or the biological activity of pyrrolotriazinones. For instance, a SciFinder^®^ search for pyrrolo[1,2-d][1,2,4]triazin-4(3H)-one (A) returns 8 references only. Herein, we focus on pyrrolotriazinones (A) and (C) published or patented to date and present their biological activities as well as their targets when available.

## 2. Pyrrolotriazinones and Their Potential Therapeutic Targets

### 2.1. Pyrrolo[1,2-d][1,2,4]triazin-4(3H)-one (A)

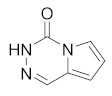



**Figure 2:**
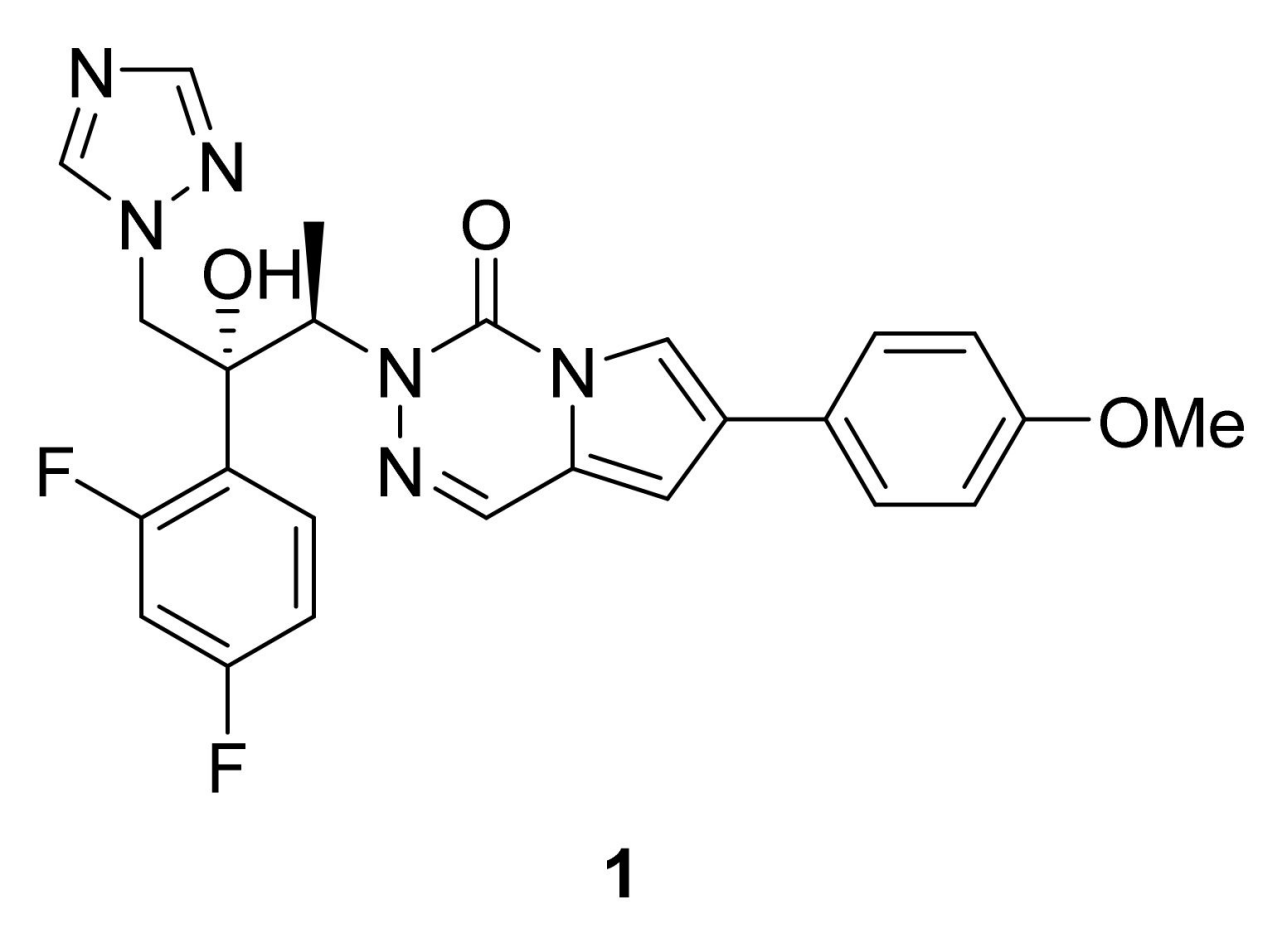
Compound **1**, antifungal agent.

Invasive fungal infections are one of the main concerns in immunocompromised patients. While azoles are the first line therapy in treating candidiasis, side effects such as liver toxicity and appearance of resistance highlight the need for new drugs. In this context, compound **1** (Figure 2), a triazole derivative bearing a pyrrolotriazinone scaffold, has shown broad in vitro antifungal activity [7]. The main biological activity may be due to the azole ring, but one cannot exclude the pharmacophoric influence of the pyrrolotriazinone ring in the inhibition of CYP51. In particular, compound **1** is active in vitro against pathogenic *Candida* spp. including fluconazole resistant strains as well as *Candida albicans* (MIC < 0.01 μg mL^−1^). In addition, the same compound is also active against some filamentous fungi such as *Aspergillus fumigatus*. Finally, the title compound also demonstrates promising in vivo activity (15 mg/kg) in murine models of lethal systemic infections caused by *Candida albicans* (Figure 2).

### 2.2. Pyrrolo[2,1-f][1,2,4]triazin-4(3H)-one (C)

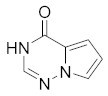



#### 2.2.1. CRF1 Receptor Antagonists

Corticotropin-releasing factor (CRF), also known as corticotropin-releasing hormone (CRH), is a peptide hormone that plays a key role in the regulation of the hypothalamic-pituitary-adrenal (HPA) axis, with the secretion of two hormones, ACTH, produced by the pituitary gland, and glucocorticoids, produced by the adrenal gland, coordinating endocrine, behavioral and autonomic responses to stress. Indeed, these hormones are increased during stress and are responsible for well-known symptoms, such as stomachaches, nausea, confusion, fatigue, etc. However, in the long term, the damages caused can be much more important, with endocrine (diabetes, hyperthyroidism, hormone deregulation), metabolic, and cardiovascular, as well as psychic, disorders. Although many treatments are already available on the market, the discovery of new potential targets and new treatments remains essential.

Preclinical and clinical studies have suggested that CRF1 antagonists could be promising in the treatment of stress-related disorders. Some studies have also shown the positive impact of these antagonists on intestinal disorders such as irritable bowel syndrome [9,10].

**Figure 3:**
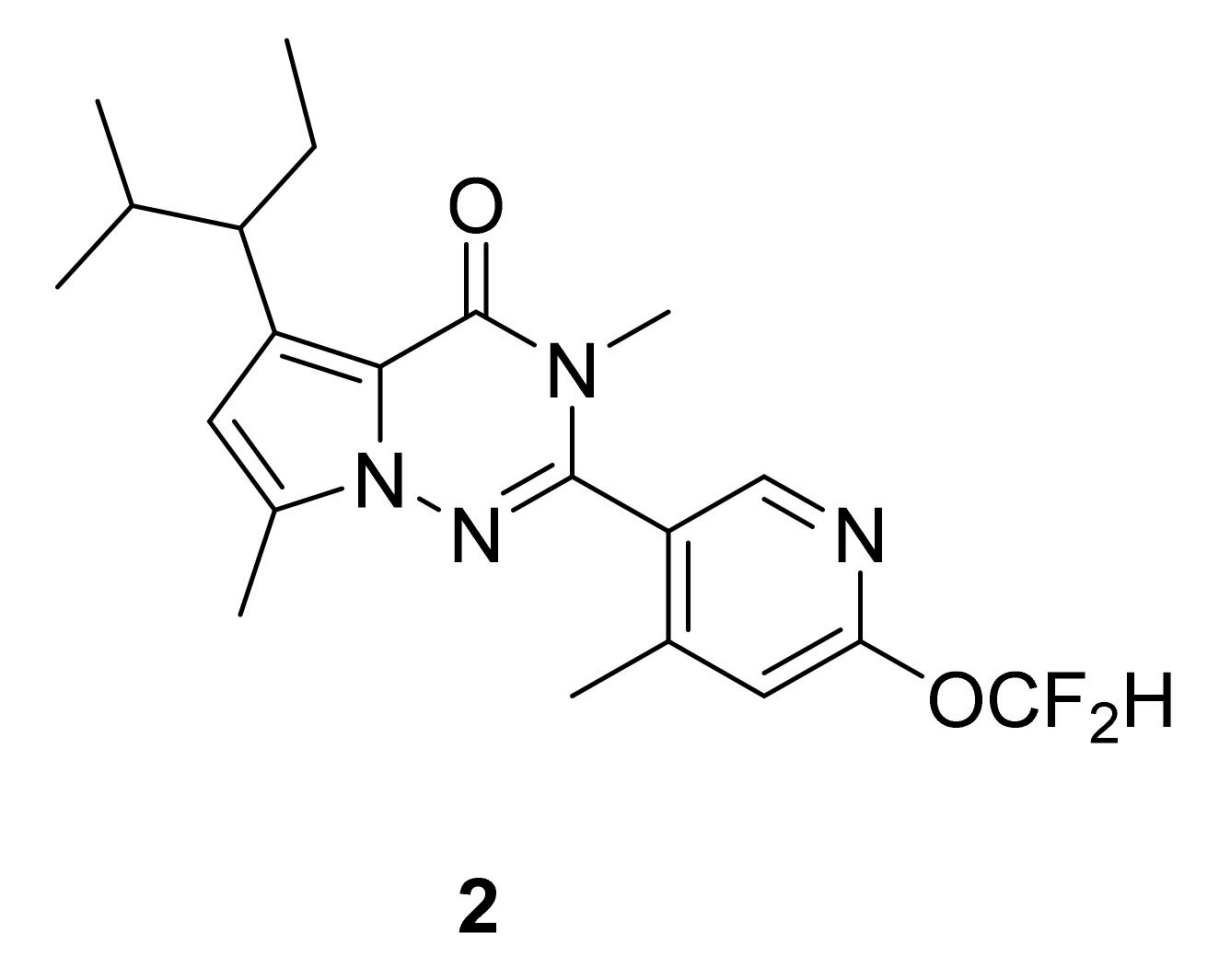
Compound **2**, CRF1 receptor antagonist.

Within this context, and through numerous investigations of structure–activity relationships, Saito et al. [11] have designed a series of pyrrolotriazinone (pyrrolo[2,1-f]triazin-4(3H)-ones) analogues by optimizing binding affinity, antagonistic activity and stability. Compound **2** (Figure 3) was the most interesting, showing very good binding affinity (IC_50_: 5.3 nM) and antagonistic activity on the CRF1 receptor (EC_50_: 3.2 nM). It has also been tested for its effect on anxiety using rat elevated plus maze test, showing efficacy in a dose–response manner.

#### 2.2.2. MCHR1 Antagonists

Melanin-concentrating hormone is a hypothalamic neuropeptide and is involved in the neural regulation of food intake and energy homeostasis.

Indeed, acute intracerebroventricular injections of MCH have shown a significant increase in food consumption, and its chronic administration significantly increased food intake, body weight, white adipose tissue mass and liver mass. Moreover, mice that lacked the pro-hormone became hypophagic and lean, and in contrast, mice overexpressing MCH became obese [12]. MCH is also thought to have other roles, notably in stress, anxiety and depression. However, it has so far only been envisioned as a target in the discovery of drugs against obesity.

**Figure 4:**
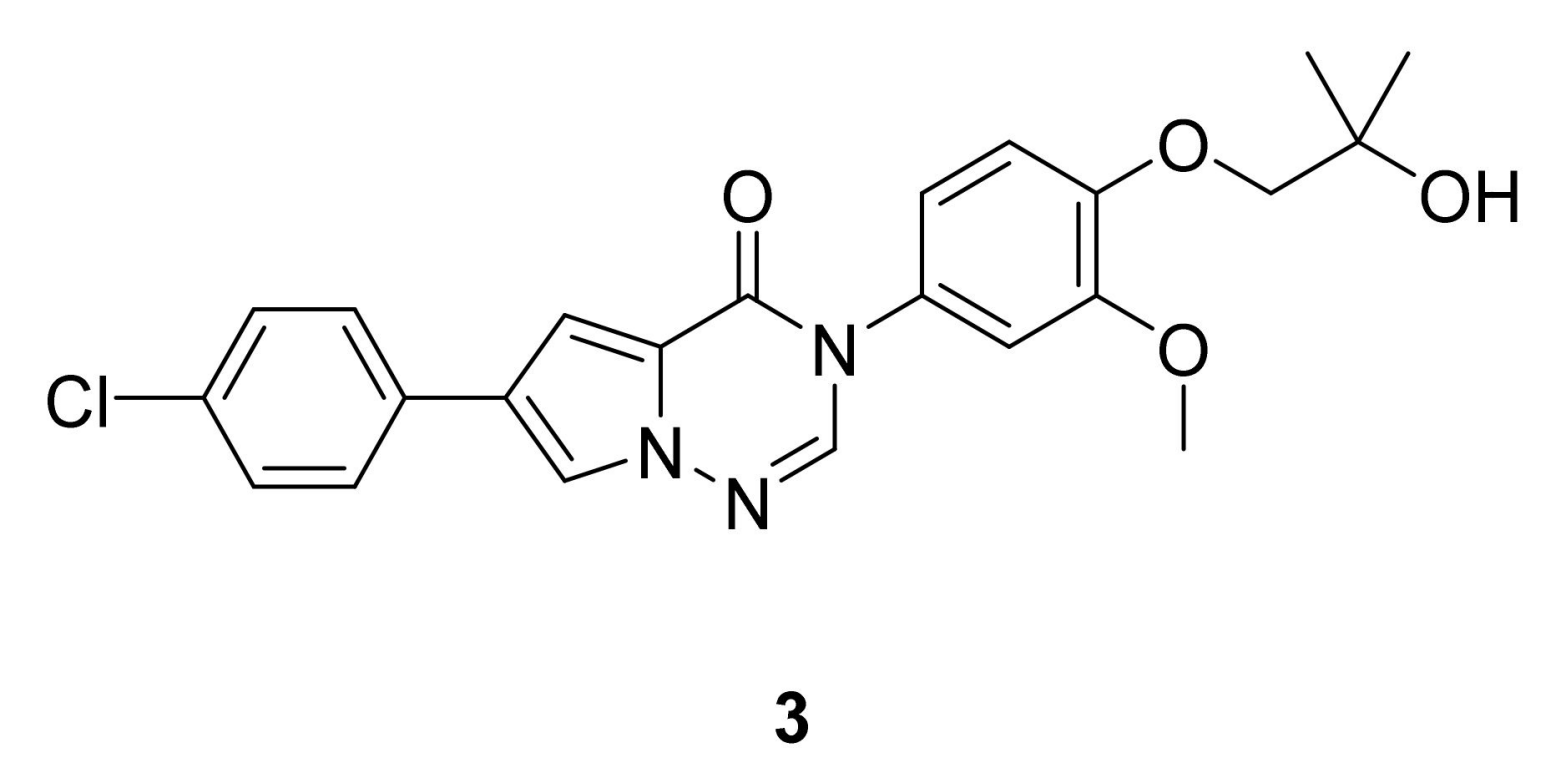
Compound **3**, MCHR1 antagonist.

Devasthale et al. [13] have designed a series of pyrrolotriazinones that were assayed for their antagonistic activity, and the reduction in weight gain in a rat model after 4 days of once-daily oral treatment. Among the tested compounds that exhibited the best brain concentrations, compound **3** (Figure 4) showed the best results, with an MCHR1 inhibition constant (Ki) of 2.3 nM (human) and 1.8 nM (rat) and an impressive 6.9% weight reduction in rat model relative to vehicle after 4 days.

#### 2.2.3. EP3 Receptor Antagonists

EP3, also known as the prostaglandin receptor EP3, is a receptor for prostaglandin E2 (PGE2) and other prostanoids whose binding induces numerous and diverse cellular responses both physiological and pathological. One can mention the stimulation of digestive secretion; the regulation of blood pressure; the inflammation mechanism, due to the presence of these receptors on platelets [14,15]; the increase of arterial thrombosis, particularly at the level of atherosclerotic vessel walls via their activation and aggregation; and, therefore, an increase in the risk of myocardial infarction and stroke.

In addition, several teams have shown the increased concentration of PGE2 in atherosclerotic vascular walls; therefore, targeting EP3 receptors could reduce thrombosis specifically in atherosclerotic vessels without increasing the risk of bleeding in healthy vessels, which is the main issue with current antithrombotic treatments [16,17,18].

**Figure 5:**
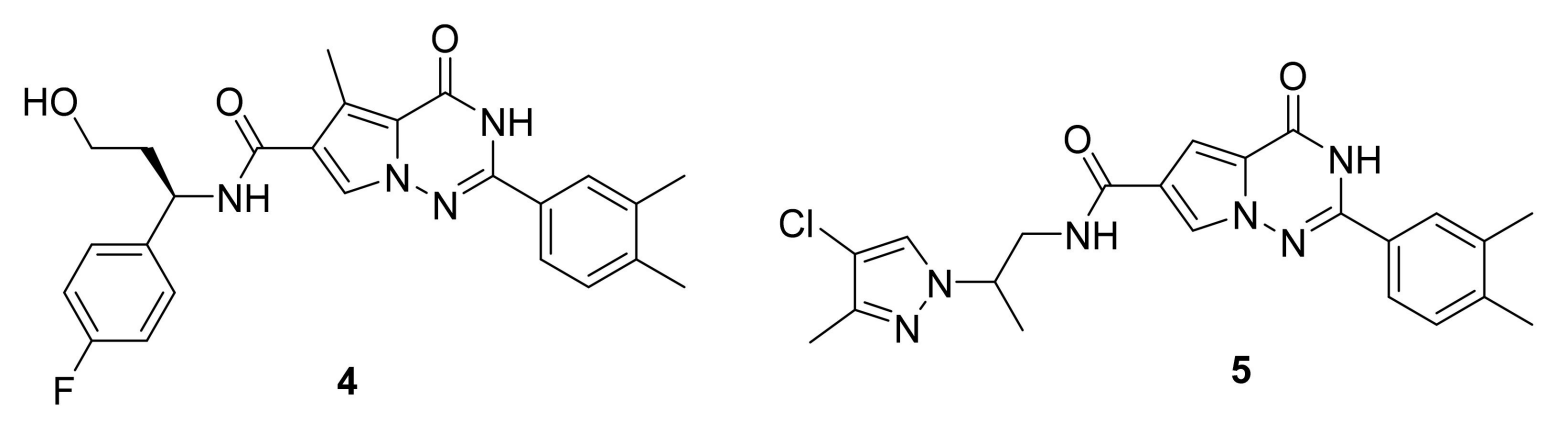
Compound **4** and **5**, EP3 receptor antagonists.

Candish et al. [19] have synthetized a series of pyrrolotriazinone carboxamide and evaluated their EP3 antagonistic ability. Many compounds showed excellent activity, including compounds **4** and **5** yielding an IC_50_ of 0.3 nM (Figure 5). These compounds’ efficacy was also tested in vivo on mice with laser injury-induced thrombi in atherosclerotic micro vessels.

#### 2.2.4. PI3K Inhibitor

Phosphoinositide 3-kinases (PI3Ks), also called phosphatidylinositol 3-kinases, are a family of lipid kinases. They are divided into class I, class II, and class III. Class I is the most extensively studied and the focus of numerous drug discovery efforts. These PI3Ks are activated by cell surface receptors to produce phosphatidylinositol-3,4,5-trisphosphate (PIP3). PIP3 is a key cellular mediator that activates downstream signaling pathways involved in relevant cellular processes such as growth, proliferation, chemotaxis, metabolism, and survival.

Class I PI3K is further subdivided into four different isoforms: PI3Kα, PI3Kβ, PI3Kδ, and PI3Kγ. The first two isoforms are ubiquitously expressed and play key roles in cell growth, survival, and proliferation; thus, inhibition of PI3Kα and PI3Kβ is primarily aimed in cancer therapy [20,21]. In the other part, PI3Kγ is widely expressed in granulocytes, monocytes and macrophages, whereas the PI3Kδ isoform is also found in B and T cells. They play a role in the production of cytokines induced by T cell receptors, the degranulation of basophils and mast cells, and the oxidative explosion of neutrophils. Thus, PI3Kδ or PI3Kγ isoform-specific inhibitors may have therapeutic benefits in auto-immune diseases, lymphoma, leukemia, and some inflammatory diseases.

**Figure 6:**
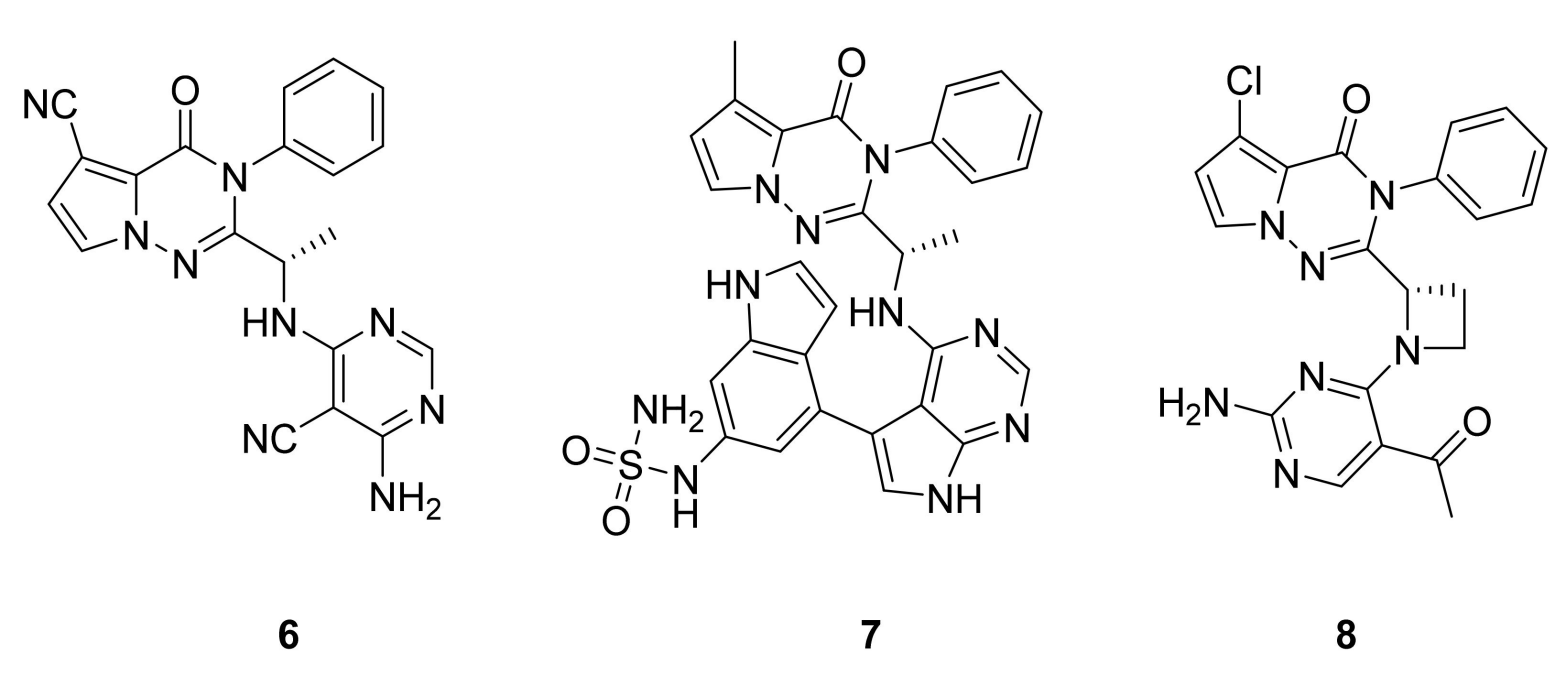
Compounds **6**–**8**, PI3K inhibitors.

Erra et al. [22,23] have studied PI3Kδ inhibition by the addition of pyrimidine and purine derivatives to the pyrrolotriazinone scaffold and, through an extensive structure–activity relationship study, shown that the compounds had a propeller-shaped conformation where the pyrrolotriazinone moiety was sandwiched into the PI3Kδ hydrophobic binding pocket between Trp760 and Met752. They observed that orally administered compound **6** (LAS191954) has good inhibitory potency (Figure 6), with a PI3Kδ IC_50_ value of 2.6 nM and very low in vitro metabolism, and is thus indicated for the treatment of inflammatory diseases. They also identified compound **7** (LAS195319), which is administered by inhalation with a PI3Kδ IC_50_ of 0.5 nM for the treatment of respiratory diseases with an inflammatory component. Jia et al. [24] have attempted to discover and optimize potent and highly selective PI3Kγ−PI3Kδ dual inhibitors (compound **8**, PI3Kγ IC_50_: 4 nM and PI3Kδ IC_50_: 5 nM, Figure 6). A docking model of the binding site of PI3Kγ showed a similar hydrophobic pocket where the pyrrolotriazinone moiety is sandwiched, formed by Met804, Ile963, and Met953. However, no biological activity study has been reported by Jia’s group, but a higher activity with this double inhibition than with the single inhibition of PI3Kδ is expected.

#### 2.2.5. Eg5 Inhibitor

Eg5, or kinesin-5, is an essential molecular motor protein in the mechanism of mitosis. Indeed, its presence allows centrosome separation, which is essential for bipolar spindle formation and sister chromatid segregation. Overexpression of Eg5 can lead to the development of spindle defects and thus to genetic instability and tumors.

On the other hand, its inhibition induces the formation of an aberrant mitotic spindle and then the arrest of the cell cycle at M phase, which leads to apoptosis of the cells and thus to the inhibition of cell proliferation It is therefore conceivable to use it in the treatment of certain human cancers [25]. Furthermore, due to a generically low expression of Eg5 in non-cancerous tissues, a therapy targeting Eg5 will be significantly less toxic compared to current anti-mitotic therapies.

**Figure 7:**
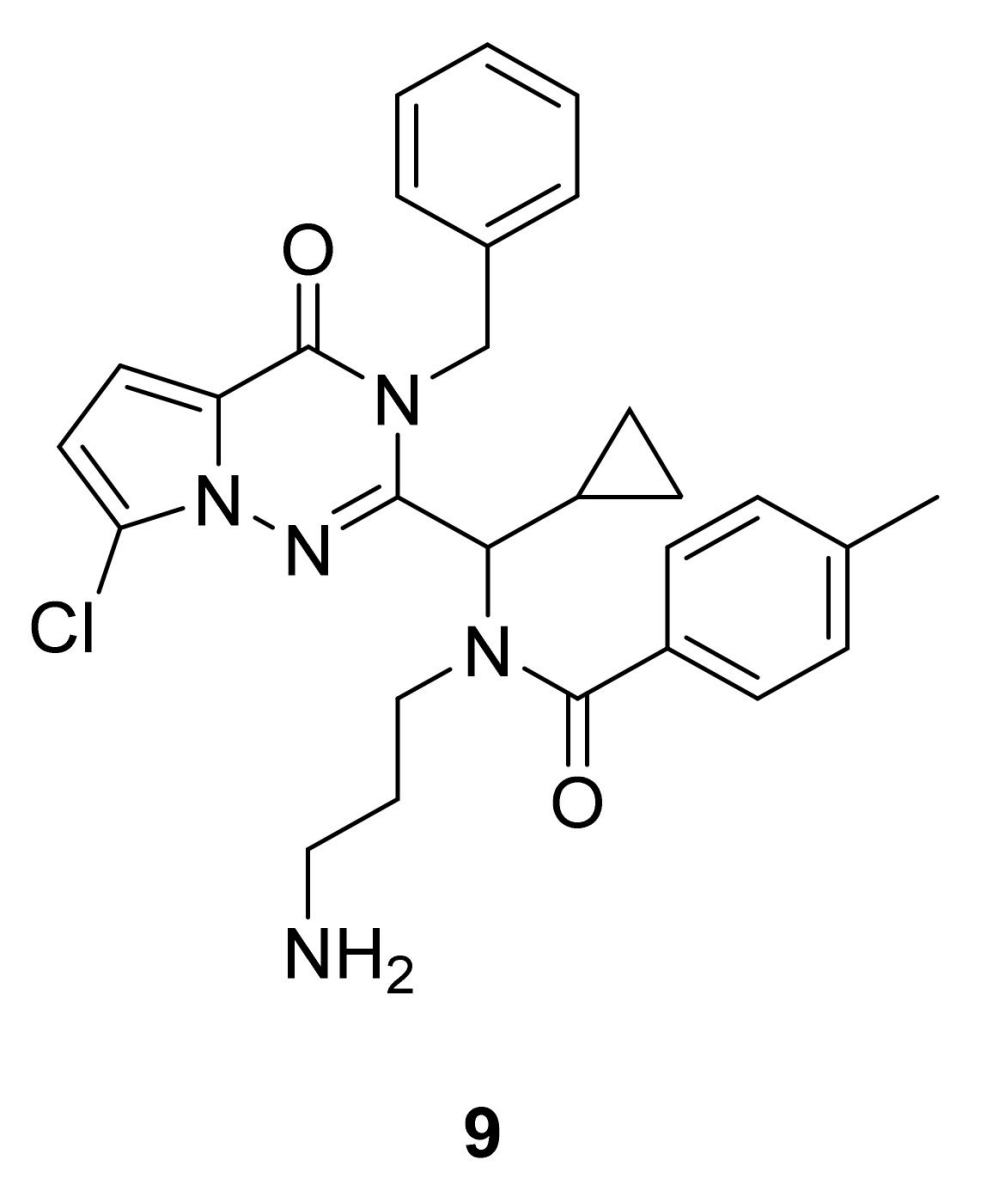
Compound **9**, Eg5 inhibitor.

Kinesin-5 motors are phosphorylated at multiple sites within the protein, and this phosphorylation impacts motor activity, localization, and function in mitosis. Thus, Kim et al. [26] have performed a structure–activity relationship study and synthesized compound **9**, which showed potent inhibition of Eg5 ATPase (IC_50_ of ATPase activity ~60 nM) and in vivo efficacy in the P388 murine leukemia model. An X-ray crystal structure of the Eg5 binding site showed a hydrophobic pocket formed by Ile136, Leu214, Phe239, and Leu160 of the protein that allows van der Waals interaction with the pyrrolotriazinone moiety, and hydrophobic interactions between the phenyl and cyclopropyl groups of **9** (Figure 7) with Trp127, Tyr211, Pro137, and Arg119 of the protein.

#### 2.2.6. Sepiapterin Reductase Inhibitors

Sepiapterin reductase plays an important role in the biosynthesis of tetrahydrobiopterin (BH4), which is a key cofactor for a set of enzymes, including nitric oxide synthases (NOSs), aromatic amino acid hydroxylases, and alkylglycerol monooxygenase. Consequently, BH4 is associated with various physiological and pathological biological processes, including monoamine neurotransmitter formation, immune response, cardiovascular function, endothelial dysfunction, and cancer.

Moreover, increased levels of BH4 in injured sensory neurons and inflamed tissues correlate with pain intensity, and its reduction by treatment with RPD inhibitors results in a decrease in pain and inflammation [27]. In a context where therapeutic options for chronic pain are scarce and almost limited to opioids, the discovery and development of new targets is relevant

**Figure 8:**
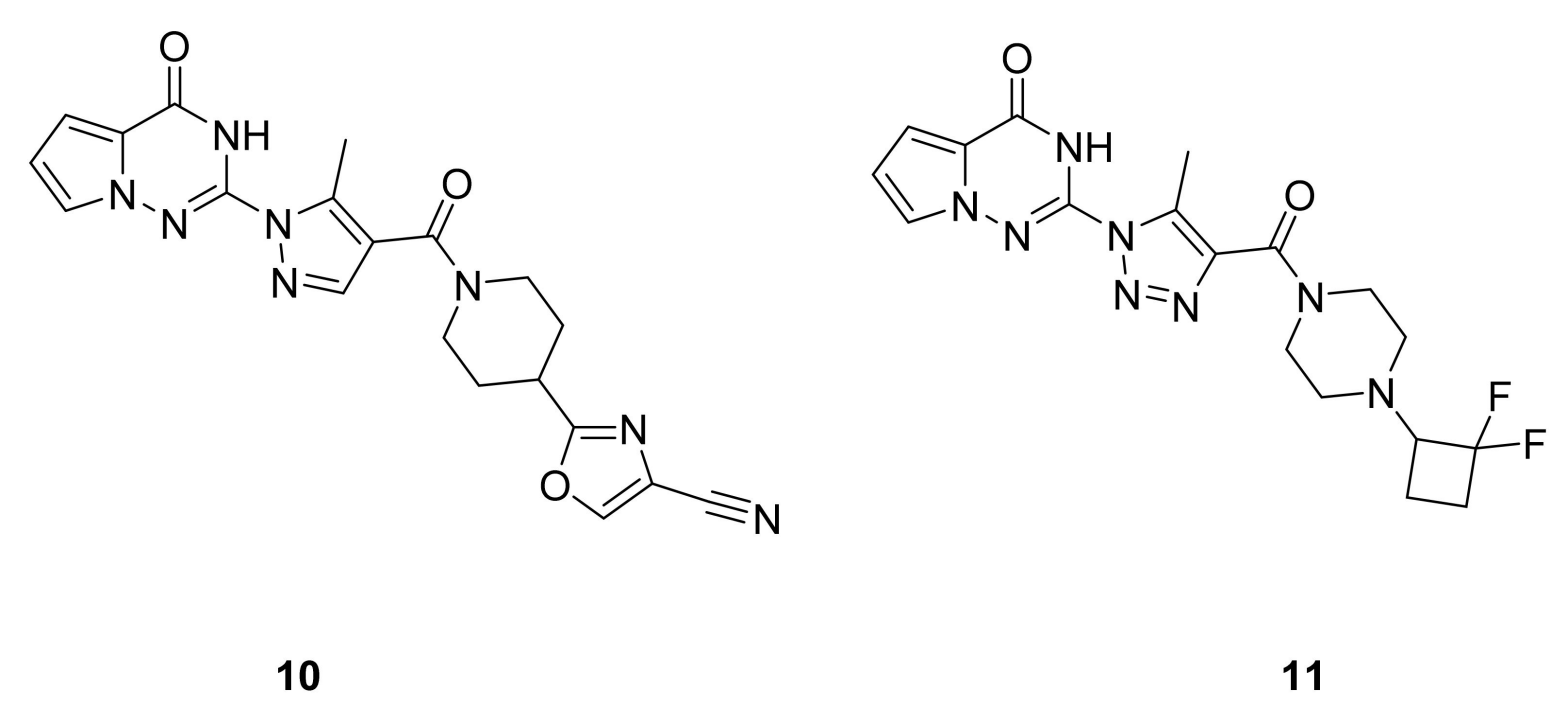
Compounds **10** and **11**, sepiapterin reductase inhibitors.

Tebbe et al. [28] have synthesized a series of pyrrolotriazinone derivatives and screened for their inhibitory activity on SPR. Many of these derivatives have shown very good activity, including compounds **10** and **11** (IC_50_: 1 nM for both compounds, Figure 8), and significantly reduce pain in behavioral pharmacology models for pain in rats.

#### 2.2.7. Ubiquitin-Specific Protease 7 Inhibitors

Ubiquitin-specific protease 7, USP7, is a de-ubiquitin enzyme that catalyzes the removal of ubiquitin from specific target proteins. Given the important role of ubiquitination of proteins, in particular for their degradation and thus their regulation, one can understand the interest in developing inhibitors for these enzymes to treat various pathologies. The involvement of USP7 in the modulation of the p53 signaling pathway has led to an increase in USP7 inhibitors, potential candidates for the treatment of cancers and immunological disorders [29].

**Figure 9:**
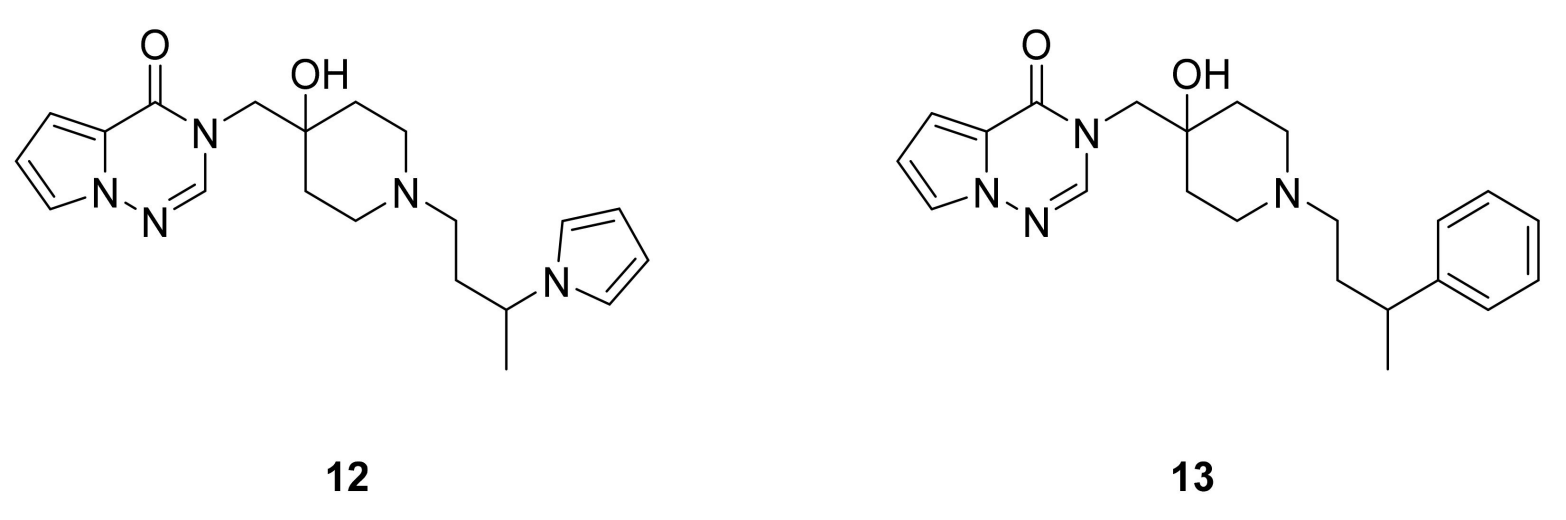
Compounds **12** and **13**, USP7 inhibitors.

Ioannidis et al. [30] have reported a series of pyrrolotriazinones and imidazotriazinones and tested their inhibitory activities against USP7. Among the synthesized compounds, **12** and **13** were the most active, with IC_50_ values below 200 nM (Figure 9).

#### 2.2.8. Stearoyl-CoA Desaturase Inhibitors

Stearoyl-CoA desaturase (Δ-9-desaturase, SCD1) is a lipogenic enzyme of the endoplasmic reticulum that catalyzes the formation of monounsaturated fatty acids, primarily oleate and palmitoleate, two major components of the biological membrane, high levels of which have been found in transformed cells and cancer tissues.

It is well understood that the degree of unsaturation and the length of the fatty acid chains of the membrane lipids influence in an important way the fluidity of biological membranes. Consequently, an imbalance of this sort, due to a modification of the ratio between saturated fatty acids and monounsaturated fatty acids, leads to an alteration of the functioning and the structure of the cell, responsible for numerous and diverse pathologies. Indeed, SCD overexpression is implicated in metabolic diseases such as diabetes, obesity, insulin resistance, hypertension, and hypertriglyceridemia as well as in various human cancer cells. Thus, SCD1 could be a potential target for anticancer and metabolic disease therapies [31,32].

**Figure 10:**
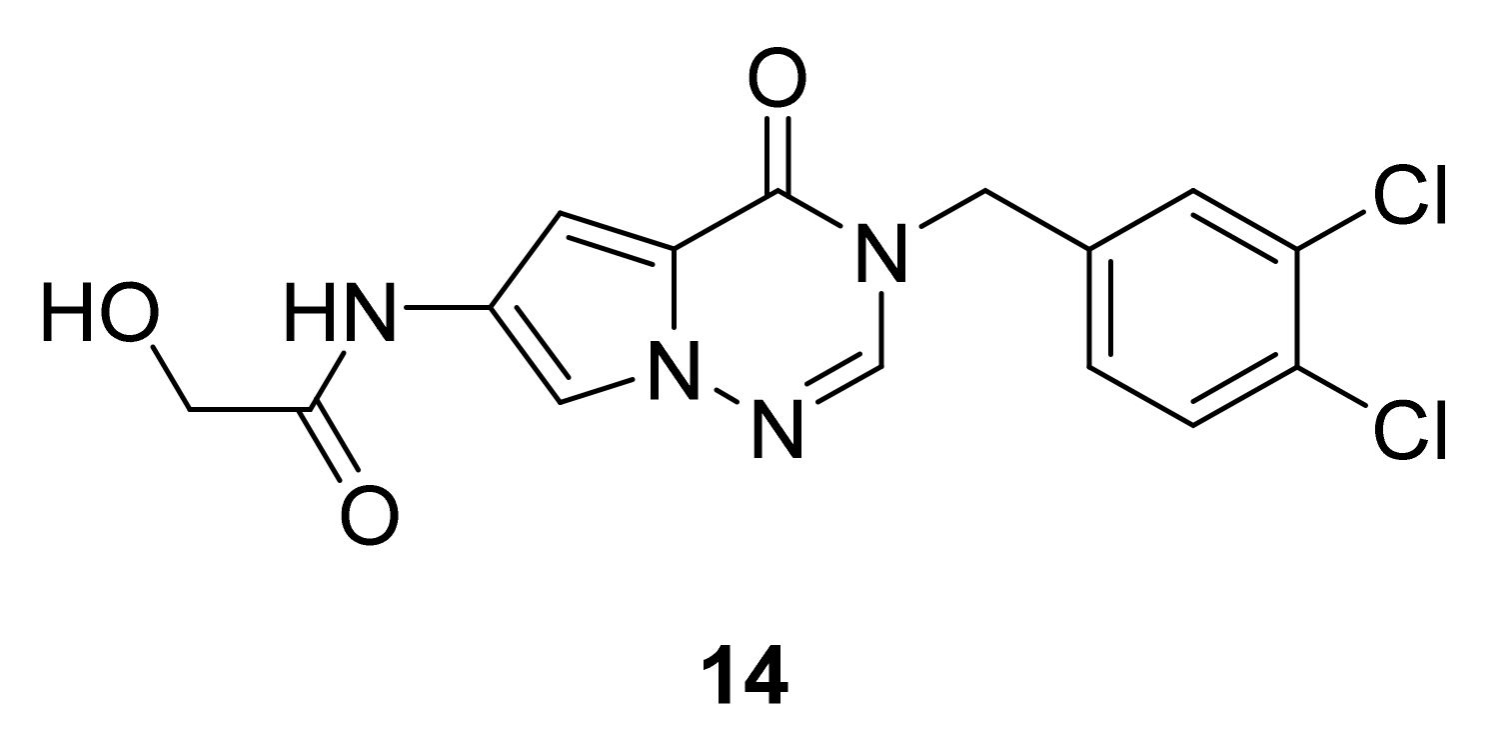
Compound **14**, stearoyl-CoA desaturase inhibitor.

Koltun et al. [33] have synthesized a series of pyrrolotriazinones and report that compound **14** has the best inhibitory activity, with an IC_50_ of 250 nM (Figure 10).

#### 2.2.9. Dipeptidyl Peptidase IV Inhibitors

DPP IV is an enzyme found on the surface of most cells and is responsible for the degradation of the incretin peptide hormones regulating blood glucose levels. DPP-IV inhibitors, known as gliptins, are a class of oral antidiabetic for the treatment of type 2 diabetes in adults. Their mechanism of action and proof of concept are well described in the literature [34].

**Figure 11:**
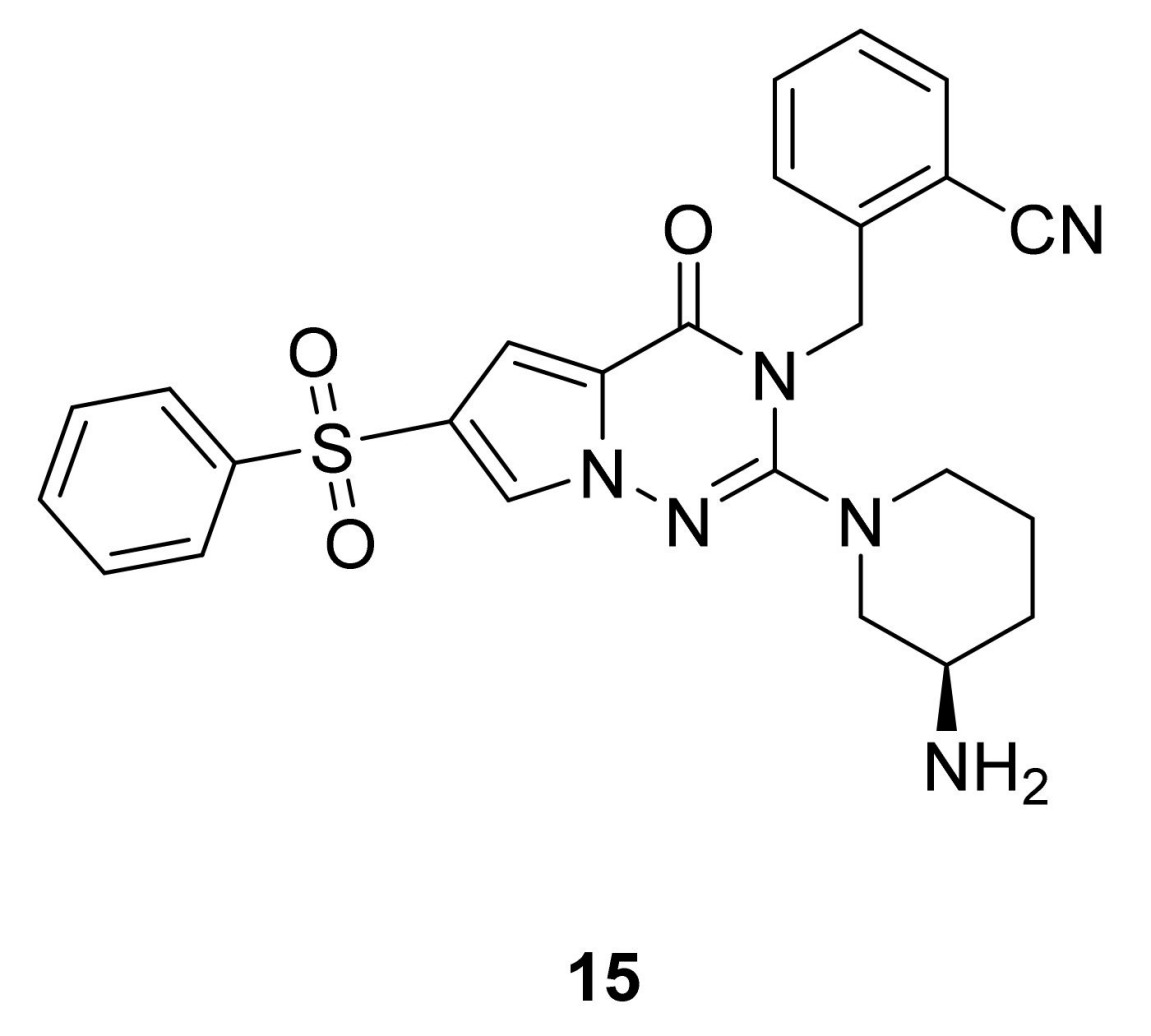
Compound **15**, dipeptidyl peptidase IV inhibitor.

Zhao et al. [35] have synthesized a series of pyrrolotriazinones and screened their activity as dipeptidyl peptidase-IV inhibitors for the treatment of diabetes. Compound **15** (Figure 11) exhibited excellent activity with an IC_50_ value of 3.5 nM and was tested in vivo in a mouse model, showing a significant decrease in blood glucose levels similar to sitagliptin compared to the negative control.

#### 2.2.10. PDE-5 Inhibitors

Phosphodiesterase-5 is an enzyme found primarily in the smooth muscle of the corpus cavernosum that selectively cleaves and degrades cGMP to 5′-GMP. The accumulation of cGMP leads to smooth muscle relaxation in the corpus cavernosum and increased blood flow to the penis, and thus erection [36]. PDE-5 inhibitors have been approved as treatment for erectile dysfunction (tadalafil, sildenafil).

**Figure 12:**
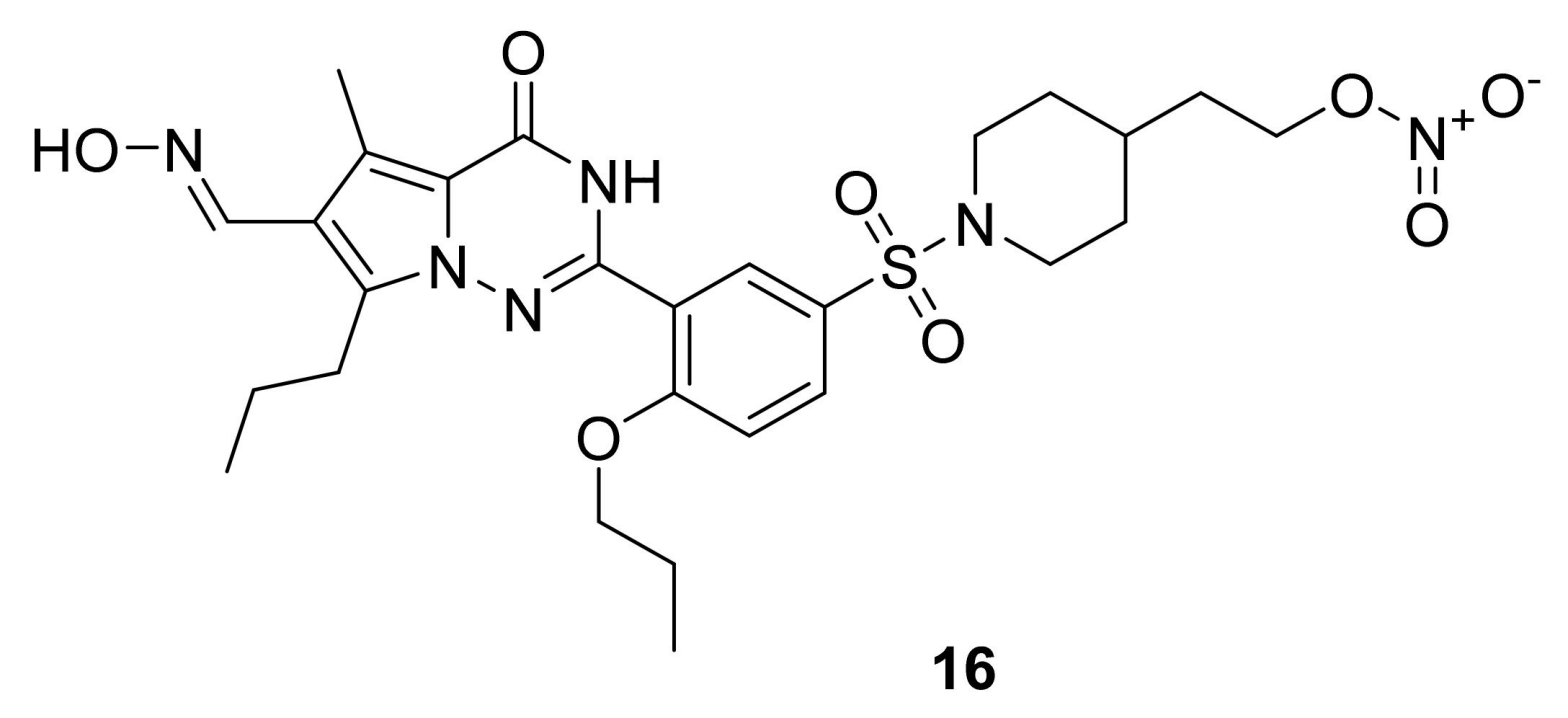
Compound **16**, phosphodiesterase-5 inhibitor.

Naef et al. [37] have designed a series of 2-phenyl-3,4-dihydropyrrolo[2,1-f] [1,2,4] triazinones and evaluated their PDE-5 inhibitory activity, and one of the products, compound **16** (Figure 12), showed an IC_50_ of 4.1 nM, which was compared to the IC_50_ of sildenafil, 5.4 nM. This compound also exerted an approximately 17-fold more potent relaxation of PE-precontracted rat aortic rings. Naef and colleagues further studied this compound and showed that it had a unique dual activity, acting as a nitric oxide donor and a phosphodiesterase 5 inhibitor. This dual activity gives it therapeutic potential for the treatment of chronic wounds [38].

#### 2.2.11. Phosphodiesterase 9 Inhibitors

Phosphodiesterase-9 is like PDE-5, an enzyme that converts cGMP (active form) to 5′-GMP (inactive form). PDE-9 inhibition is required to treat diseases that lower the level of cGMP. PDE-9 is expressed mainly in the brain, kidneys, spleen, small intestine, and blood cells and is associated with disorders such as CNS diseases, sleep disorders, schizophrenia, cardiovascular diseases, insulin-resistance syndrome and diabetes, and sickle cell disease [39].

**Figure 13:**
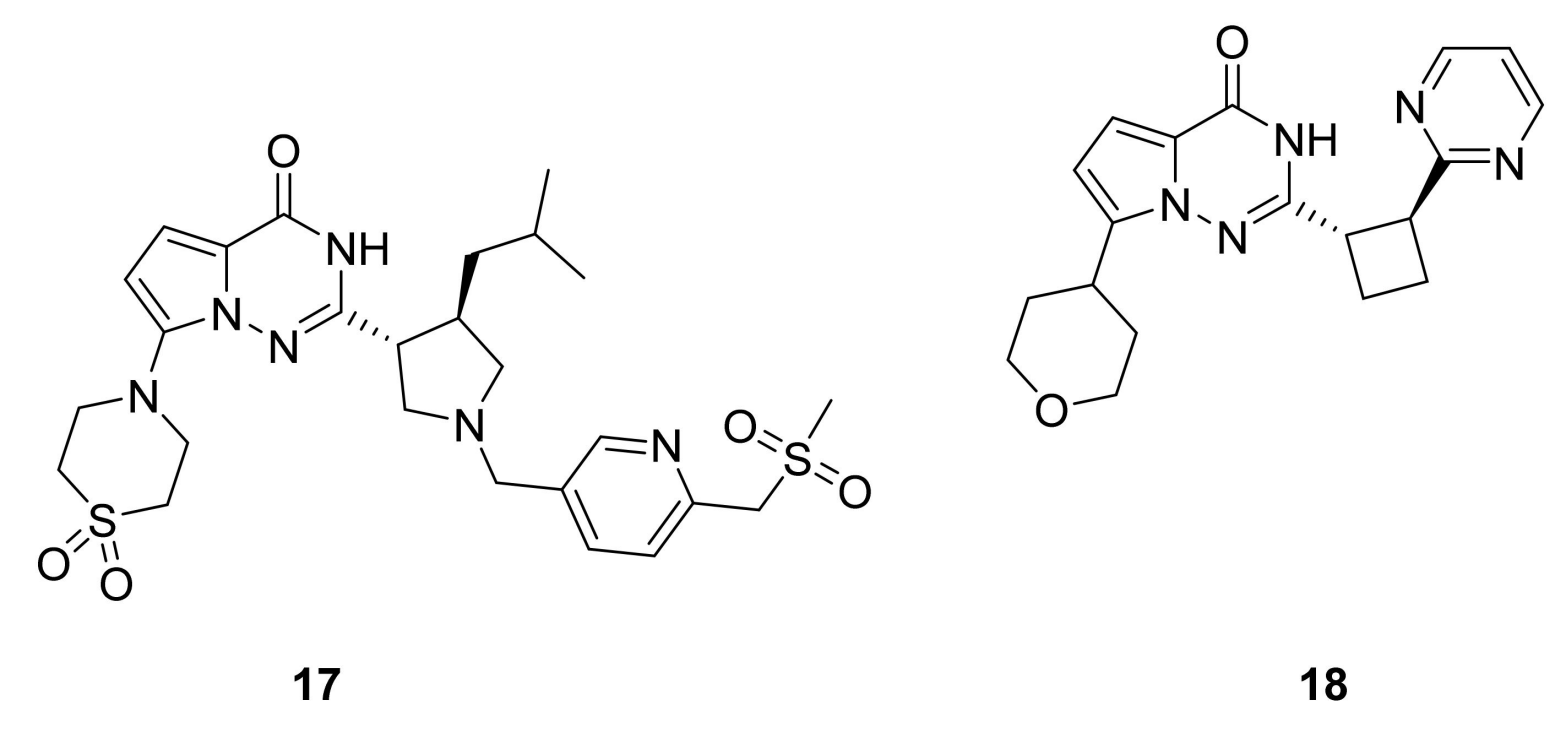
Compounds **17** and **18**, phosphodiesterase-9 inhibitors.

Li and others [40,41] have synthesized a series of pyrrolotriazinones and tested their PDE-9 inhibitory activity. Compounds **17** and **18** (Figure 13) have shown good activity (IC_50_: 2 nM and 10 nM, respectively).

Shao et al. [42] studied the PDE-9 binding site, which has a small pocket for the pyrrolotriazinone moiety formed by Gln453, which allows two hydrogen bonding, and by Phe456, which allows aromatic π-stack; both of these are two characteristic interactions of inhibitors with various PDE families

#### 2.2.12. Tankyrase and Wnt Pathway Inhibitors

The WNT/catenin signaling pathway plays critical roles in the context of cell development, differentiation, and self-renewal. Thus, overactivation of WNT/β-catenin signaling is implicated in various types of cancers by leading to increased proliferation. As a major activator of the WNT/β-catenin pathway in colon cancer, tankyrase appears to be a good target for anticancer drug development. Abnormal activation of tankyrase stabilizes cellular β-catenin through degradation of axin proteins, which are the crucial components of the β-catenin destruction complex. Tankyrases belong to the family of proteins known as poly (ADP)-ribose polymerases (PARPs) and consist of two members: tankyrase 1 (TNKS-1) and tankyrase 2 (TNKS-2) [43].

**Figure 14:**
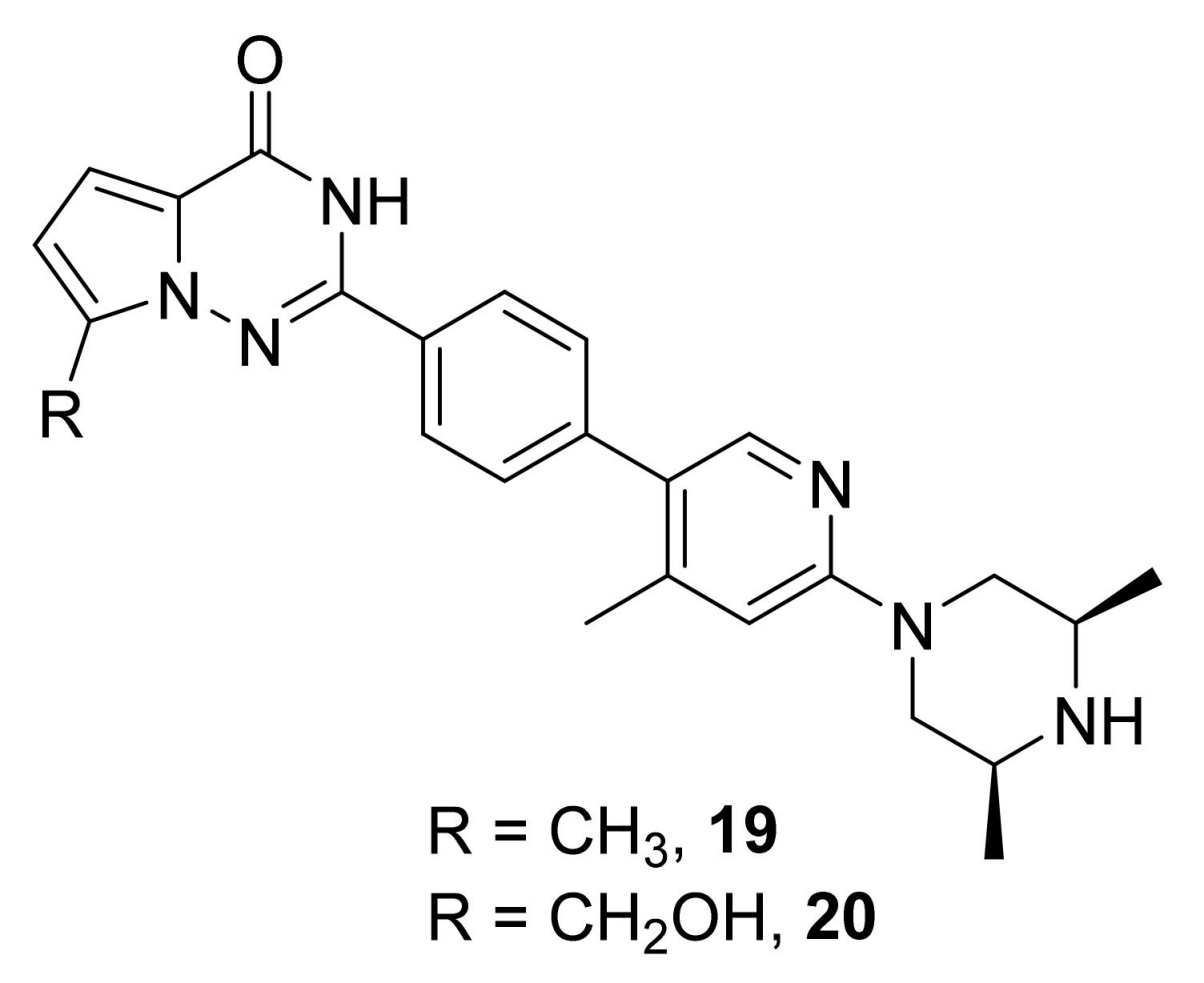
Compounds **19** and **20**, tankyrase and Wnt pathway inhibitors.

Johannes et al. [44] have synthesized a series of pyrrolotriazinone derivatives and evaluated their inhibitory potency on TNKS-1 and TNKS-2 as well as on Wnt pathway inhibition in DLD-1 cells. An X-ray crystal structure of one the compounds bound to TNKS1 showed that the pyrrolotriazinone ring is sandwiched between Tyr1224 and Tyr1213 of TNKS1, making π-stacking interactions. Two compounds, **19** and **20** (Figure 14), have shown acceptable potency (TNKS-1 IC_50_ values: 8 nM (**19**) and 4 nM (**20**); Wnt cell IC_50_ values: 3 nM (**19**) and 12 nM (**20**)).

## 3. Conclusions

In conclusion, as with other nitrogen-based heterocycles, the pyrrolotriazinone core may represent a relevant starting scaffold for drug design. Whereas very few biological activities of pyrrolotriazinones have been described so far, there is no doubt that new compounds of this chemical family will be identified as leads and may reach the market as therapeutic agents in the future. To date, the biological activities refer to inhibition of a few enzymes, but also to antagonists of a few receptors. Many other biological targets no doubt remain to be discovered for pyrrolotriazinones.

## Data Availability

Data sharing is not applicable.
